# Impact of Japanese Subpopulation Data and Overall Survival in Pivotal Trials on Oncology Drug Approvals in Japan

**DOI:** 10.1007/s43441-026-00970-8

**Published:** 2026-05-12

**Authors:** Takanobu Watanabe, Ikuo Sugiyama, Hideyuki Kondo

**Affiliations:** Regulatory Affairs, Gilead Sciences K.K., GranTokyo South Tower, 1-9-2 Marunouchi, Chiyoda-ku, Tokyo, 100-6614 Japan

**Keywords:** Japanese population, MRCT, Oncology, Overall survival, PMDA

## Abstract

Globalization of oncology drug development has accelerated the adoption of multiregional clinical trials (MRCTs) as pivotal evidence for regulatory approvals in major markets, including Japan. While MRCTs enhance efficiency and generalizability, they introduce challenges related to inter-ethnic variability and data extrapolation. Japan’s regulatory framework, guided by pharmaceuticals medical devices agency (PMDA) principles and the “Guideline for Clinical Evaluation Methods of Anticancer Drugs,” emphasizes patient efficacy, safety and relevance, historically requiring local data to supplement global findings. This review analyzed the approvals of oncology drugs in Japan over 5 years, focusing on the role of Japanese subgroup data and overall survival (OS) outcomes in pivotal trials. Among 165 approvals for oncology drugs, OS in pivotal trials was set as a sole primary endpoint in 9.1% (15/165) and as co-primary/dual primary endpoints in 10.3% (17/165); alternative endpoints such as progression-free survival were accepted when OS was impractical. In 7.9% (13/165) of all approvals, Japanese subpopulation results diverged from trends of overall population (hazard ratio [HR] > 1 in Japanese subpopulation), and 4.2% (7/165) of the approvals involved trials in which the primary endpoint of OS (including a sole, co-primary or dual primary endpoint) was not achieved. The PMDA decisions reflected holistic evaluation, considering trial design integrity, secondary endpoints, and qualitative consistency rather than rigid HR thresholds. Differences in efficacy in Japanese subgroup rarely impacted labeling, underscoring reliance on global evidence with local scientific considerations. These findings highlight the PMDA’s pragmatic approach to endpoint flexibility and extrapolation, reinforcing the importance of early regulatory engagement and strategic alignment in MRCT design for oncology drug development in Japan.

## Introduction

The rapid globalization of oncology drug development over the past two decades has catalyzed the emergence of large-scale, multiregional clinical trials (MRCTs) as the primary evidence base for regulatory approvals in major markets, including the United States, European Union, and Japan [1, 2]. This shift is driven by the imperative to expedite patient access to innovative therapies, leverage diverse patient populations, and streamline the drug development process. MRCTs facilitate robust statistical analyses and generalizability; however, they also pose challenges related to inter-ethnic variability, healthcare infrastructure, and medical practice patterns. In oncology, such variability is particularly pronounced, owing to genetic, environmental, and social determinants impacting disease biology, pharmacokinetics, and patient outcomes.

Japan, with its unique demographic profile and healthcare system, has historically exhibited cautious yet adaptive regulatory practices. The Pharmaceuticals Medical Devices Agency (PMDA) operates under principles that prioritize patient safety, efficacy, and relevance of Japanese patients, often requiring additional local data to supplement global trial findings. For drugs subject to approval review in Japan, efficacy and safety are generally evaluated based on the submitted pivotal trial, and the efficacy in Japanese patients is systematically evaluated based on the formal regulatory notifications [3–6]. Although a new guidance was issued recently that does not require conduct of a Phase 1 trial in Japanese patients before initiating an MRCT if drugs in development can be judged to be clinically acceptable, the essential need to ensure the safety and tolerability of Japanese patients remains the same even before and after the issuance of the guideline [7]. This stance reflects both historical precedent and ongoing concerns about the applicability of foreign data to Japanese patients—concerns that are magnified in oncology due to the complexity of disease mechanisms and treatment responses. Regulatory guidance in Japan, such as the “Guideline for Clinical Evaluation Methods of Anticancer Drugs” [8] underscores the need for safety assurance of the Japanese population in the clinical trial setting and rigorous evaluation of endpoints like overall survival (OS) in clinical trial, but also recognizes the role of alternative measures when OS is impractical or confounded.

The rationale for population-specific data stems from several factors: documented differences in drug metabolism and response among East Asian populations, variations in standards of care, and the ethical imperative to ensure that approved therapies are both effective and safe within the domestic context. Bridging trials and local clinical trials have historically served as mechanisms for generating Japanese-specific data, although recent years have seen greater reliance on MRCTs, supported by subgroup analyses and scientific extrapolation. MRCTs, while enabling more efficient drug development and broader patient recruitment, introduce complexities in ensuring that Japanese subgroups are adequately represented and that their outcomes are interpretable within the global context.

Further, the integration of OS as a primary endpoint in pivotal trials has presented both opportunities and challenges. While OS remains the gold standard for demonstrating clinical benefit in oncology, its measurement is often complicated by factors such as post-progression therapies, crossover designs, and the lengthy natural history of certain cancers. The PMDA’s approach to evaluating OS, and its willingness to consider alternative endpoints such as progression-free survival (PFS) reflects a nuanced balance between scientific rigor and practical feasibility. This balance is increasingly relevant as novel molecularly targeted agents and immunotherapies, with unique mechanisms of action and response kinetics, enter the Japanese market.

This review seeks to systematically analyze the impact of Japanese subpopulation data and OS outcomes in pivotal trials underpinning oncology drug approvals from Fiscal Year (FY) 2020 to FY 2024, situating these findings within the broader context of global regulatory practice. By analyzing the PMDA assessment reports, we aim to elucidate trends of approval decision-making with a comprehensive understanding of the discussion in the review.

## Materials and Methods

### Data Sources

Our analysis leveraged two primary sources of regulatory information. First, we systematically extracted data from the official PMDA website [9], which maintains an up-to-date registry of new drug approvals, including details such as drug name, approval date, therapeutic indication, and approval classification (e.g., orphan designation, expedited review). Second, we obtained and reviewed the PMDA’s published new drug assessment reports [10], which offer comprehensive insights into clinical trial design, patient demographics, efficacy and safety outcomes, and the agency’s regulatory deliberations.

It should be noted that “new drug” in the PMDA website [11] and this article refers to a pharmaceutical product that is clearly different from an already approved drug in terms of active ingredient, strength, dosage form, route of administration, indications, or effects, as stipulated in Article 14 − 4 of the Pharmaceuticals and Medical Devices Act [12]. It mainly includes drugs containing new active ingredients, new combination prescription drugs, drugs with new routes of administration, drugs with new indications, drugs in new dosage forms, and drugs with a new dosage.

## Inclusion Criteria for Research

The scope of this research encompassed all oncology drugs approved by the PMDA from FY 2020 to FY 2024 (i.e., April 2020–March 2025). Inclusion criteria of drug approvals for this research were defined as follows:

- Drugs must be classified under oncology indications, including cytotoxic agents, targeted therapies, immunotherapies, and supportive care agents with anticancer claims.
- Only products with the PMDA assessment reports explicitly designating pivotal clinical trials as “evaluation data” for efficacy and safety were included. Trials cited as “reference data” or supplementary evidence were excluded from the analysis.
- Approvals were tabulated as a single approval in the analysis to avoid double counting if combination therapies were approved based on the same clinical trial results and the component drugs were submitted as separate applications. In other words, if the regimen was combination therapy (e.g., concomitant administration of Drug A and Drug B) and each of them was approved separately, it was counted as 1 approval in the analysis because it was a regulatory approval based on the same PMDA review results.

A “pivotal clinical trial” was defined as the trial or set of trials that served as the central basis for the PMDA’s determination of a drug’s efficacy and safety in the PMDA assessment report, typically corresponding to phase II/III MRCTs or registration trials submitted in the New Drug Application.

### Data Extraction and Analytical Methodology

We collected data from the list of approved new drugs on the website of the PMDA [9] and identified drugs approved from FY 2020 to FY 2024. Data extraction was performed by two independent reviewers to ensure accuracy and minimize bias. Each PMDA assessment report was systematically analyzed to identify pivotal trial characteristics, efficacy analyses, subgroup analyses, and regulatory commentary specific to efficacy results and Japanese subpopulation data. Discrepancies were adjudicated via consensus discussion with a third reviewer. The following data was collected and managed:

- Drug and indication details.
- Application classification (Initial or Partial change approval, Review process of the PMDA assessment).
- Approval date.
- Information on trials discussed as pivotal clinical trials in the PMDA assessment report.
- Number of trial participants (Overall, Japanese).
- Key efficacy results in overall population.
- Key efficacy results in Japanese subpopulation.
- Discussions and interpretations by the Applicant and the PMDA in efficacy outcomes in the PMDA assessment report.

We extracted the new drugs that did not show consistency in Japanese subpopulation, or did not demonstrate efficacy results with statistically significant in the trials, according to the definitions as below:

- Case 1: The results in Japanese subpopulation diverged from those in the overall population for the primary endpoints, with Hazard ratio (HR) > 1 in Japanese subpopulation.
- Case 2: OS results were not statistically significant in trials that designed OS as a primary or co-primary/dual primary endpoint.

As this investigation was conducted based on HRs and statistical significance as key indicators in efficacy analysis, clinical trials without a control arm were excluded from the analysis.

For Case 1, we also looked into each PMDA assessment report for the new drugs extracted to investigate discussion points between the applicant and the PMDA on the efficacy in Japanese patients, by the classifying below:

A) Imbalance of subject demographics.
B) Effect of subsequent therapy on efficacy.
C) Limited number of Japanese participants/events.
D) Efficacy claims by support data other than the primary endpoint (e.g., co-primary or secondary).
E) PK similarity between Japanese and non-Japanese.
F) Demonstration of superiority in the overall population.
G) No difference in diagnosis/treatment system between Japanese and non-Japanese.
H) No difference in efficacy between Japan and overseas for the approved indications.

We qualified the use of each opinion category (A-H) in discussions of efficacy in Japanese patients and evaluated the associated trend.

## Results

### Identification of Approved Oncology Drugs (FY 2020–FY 2024)

**Fig. 1:**
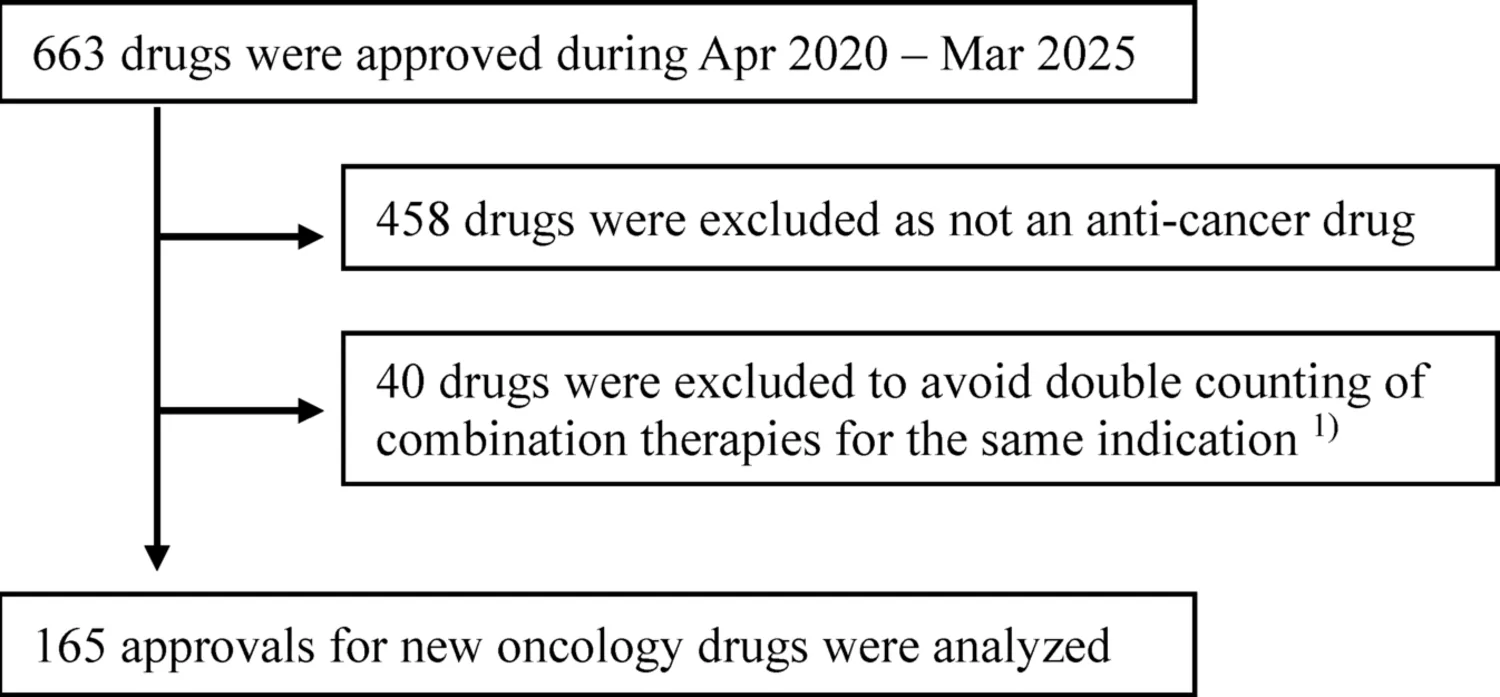
Flowchart of new oncology drug approval identification in Japan between FY 2020 and FY 2024. If the regimen was combination therapy (e.g., concomitant administration of Drug A and Drug B) and each of them was approved for the relevant indication, since the regulatory approval was based on the same PMDA review results, it was counted as 1 approval in the analysis

During the five-year period analyzed based on the PMDA’s list of new drug approvals, the PMDA approved a total of 663 new drugs, of which 205 were classified as anticancer agents. After excluding 40 drugs that were used in combination therapy for the same indication (if multiple drugs were used concomitantly and each of them was approved, it was counted as 1 approval), 165 approvals for oncology drugs were included in the analysis (Fig. 1). The composition of approved new drugs reflected the growing diversity of oncology therapeutics, with targeted agents (e.g., tyrosine kinase inhibitors, monoclonal antibodies) and immunotherapies (e.g., checkpoint inhibitors) constituting an increasing proportion of new approvals, in line with global trends (Table 1). Drugs classified as conventional chemotherapy were approved for the partial change to be used in combination with other advanced therapies.

**Table 1:** Five-year overview of approved drugs and oncology drug categories

Fiscal year	# Approved drugs	# Approved drugs for oncology	% (oncology/total)	Drug classification								
				Hormone therapy	Chemotherapy^1^	PARP inhibitor	Kinase inhibitor	mAb (non-ADC)	ADC	Bispecific Ab	Checkpoint inhibitor	Others^2^
2020	123	44	35.8%	2	5	3	11	7	3	0	9	4
2021	146	44	30.1%	0	5	0	8	5	1	0	12	13
2022	136	40	29.4%	1	10	1	6	2	4	0	10	6
2023	123	27	22.0%	1	5	2	5	3	3	2	4	2
2024	135	50	37.0%	0	6	1	16	1	4	6	10	6
Total	663	205	30.9%	4	31	7	46	18	15	8	45	31

Ab: Antibody; ADC: Antibody-Drug Conjugate; mAb: monoclonal Antibody; PARP: poly ADP-ribose polymerase; #: number of; %: percentage

^1^“Chemotherapy” includes only conventional chemotherapy such as traditional cytotoxic chemotherapeutic agents (e.g.,cisplatin).

^2^“Others” includes novel molecular-targeted drugs such as EZH1 and IDH1 inhibitors.

The annual breakdown of approvals demonstrated steady performance in the number of oncology drugs entering the Japanese market although the trend in FY 2023 was different from others perhaps due to the impact of COVID-19 pandemic, with a marked increase in drugs with new technologies such as bispecific antibodies have also appeared in recent years. Orphan-designated products accounted for approximately 27.8% (57/205) of oncology drugs, highlighting the PMDA’s commitment to addressing unmet medical needs. Faster review pathways, such as the Sakigake designation, Conditional early approval system, the Orphan designation, Priority review, were utilized in 39.5% (81/205) of the oncology drugs, reflecting regulatory responsiveness to advances in cancer therapeutics. However, drugs approved by public domain pathway accounted for approximately 10.7% (22/205) of the oncology drugs, highlighting the serious situation of drug lag in Japan.

### Oncology Drugs Approved with Outlier Results in Efficacy Endpoint

Of the 165 approvals for oncology drugs analyzed, 17 were met with either Case 1 or 2 as defined in the section of “Data Extraction and Analytical Methodology”.

Table 2 provides detail information on these 17 approvals with key efficacy results in the overall population and Japanese subpopulation. Twenty-five oncology drugs were contributing to monotherapy or combination therapy for the approvals.

**Table 2:** List of approved oncology drugs meeting the criteria of efficacy outcome in the research

Brand name/generic name	Application classification	Approval in	Indication	Pivotal trial in PMDA assessment report	Primary endpoints in pivotal trial	Number of trial participants	Key efficacy results in overall population [HR (95% CI; *p* value)]	Key efficacy results in Japanese subpopulation [HR (95% CI; *p* value)]
Sarclisa/Isatuximab (Genetical Recombination)	Initial	Jun-20	Relapsed or refractory multiple myeloma	EFC14335 (ClinicalTrials.gov ID: NCT02990338, Phase 3)	PFS per IRC assessment	307 (154 [including 9 Japanese] in isatuximab + Pd, vs. 153 [including 4 Japanese] in Pd)	PFS: 0.60 (95% CI: 0.44, 0.81; *p* = 0.001)	PFS: **1.2** (95% CI: 0.13, 11.8)
Keytruda/Pembrolizumab (Genetical Recombination)	Partial change (new indication, new dosage)	Aug-20	Unresectable advanced or recurrent PD-L1 positive esophageal squamous cell carcinoma that has progressed after cancer chemotherapy	KEYNOTE-181 (ClinicalTrials.gov ID: NCT02564263, Phase 3)	OS in the following population:	628 in the global cohort (314 [including 77 Japanese] in pembrolizumab, vs. 314 [including 75 Japanese] in the IC)	OS in the re-analysis:	OS in the re-analysis:
					1. PD-L1 CPS ≥ 10		PD-L1 CPS ≥ 10:	PD-L1 CPS ≥ 10:
					2. Squamous cell carcinoma		0.70 (95% CI: 0.52,0.94; *p* = 0.00855^*1^)	0.70 (95% CI: 0.43, 1.13)
					3. ITT		*Not statistically significant*	
							*1: significance level (one-sided) of 0.00853	
							Squamous cell carcinoma:	Squamous cell carcinoma:
							0.77 (95% CI: 0.63, 0.96; *p* = 0.00894^*2^)	0.68 (95% CI: 0.48, 0.97)
							***Not statistically significant***	
							*2: significance level (one-sided) of 0.0076	
							ITT:	ITT:
							0.89 (95% CI: 0.75, 1.05; *p* = 0.08431^*3^)	0.68 (95% CI: 0.48, 0.96)
							***Not statistically significant***	
							*3: significance level (one-sided) of 0.00772	
Tesentriq/Atezolizumab (Genetical Recombination)	Partial change (new indication, new dosage)	Sep-20	Unresectable hepatocellular carcinoma	IMbrave150 (ClinicalTrials.gov ID: NCT03434379, Phase 3)	PFS per IRF assessment	501 (336 [including 35 Japanese] in atezolizumab +bevacizumab, vs. 165 [including 26 Japanese] in sorafenib)	PFS:	PFS:
	*Priority Review*				OS		0.59 (95% CI: 0.47, 0.76; *p* < 0.0001)	0.85 (95% CI: 0.39, 1.86; *p* = 0.6903)
							OS in the 1st IA:	OS in the IA:
Avastin/Bevacizumab (Genetical Recombination)	Partial change (new indication, new dosage)						0.58 (95% CI: 0.42, 0.79; *p* = 0.0006)	**1.71** (95% CI: 0.50, 5.84; *p* = 0.3891)
	*Priority Review*							
Braftovi/Encorafenib	Partial change (new indication, new dosage)	Nov-20	Unresectable advanced or recurrent colorectal cancer with BRAF mutation that has progressed after cancer chemotherapy	BEACON CRC (ClinicalTrials.gov ID: NCT02928224, Phase 3)	Response rate per BICR assessment	665 in Ph3 part (224 [including 3 Japanese] in encorafenib + binimetinib + cetuximab (E/B/C), vs. 220 [including 6 Japanese] in encorafenib + cetuximab (E/C), vs. 221 [including 11 Japanese] in IC)	Response rate (E/B/C vs. IC):	Response rate:
	*Priority Review*				OS		*p* < 0.0001	NR
							Response rate (E/C vs. IC):	
							*p* < 0.0001	
							OS in the IA (E/B/C vs. IC):	
							0.52 (95% CI: 0.39, 0.70; *p* < 0.0001)	
							OS in the IA (E/C vs. IC):	OS in the IA (E/C vs. IC):
Mektovi/Binimetinib	Partial change (new indication, new dosage)						0.60 (95% CI: 0.45, 0.79; *p* = 0.0002)	1.58 (95% CI: 0.09, 27.19; p = 0.6248)
	*Priority Review*							
Venclexta/Venetoclax	Partial change (new indication, new dosage)	Mar-21	Acute myeloid leukemia	M15-656	*M15-656*	*M15-656*	*M15-656*	*M15-656*
	*Orphan drug*			(ClinicalTrials.gov ID: NCT02993523, Phase 3)	OS	433 (287 [including 24 Japanese] in the venetoclax + azacitidine, vs. 146 [including 13 Japanese] in placebo + azacitidine)	OS in the 2nd IA:	OS in the 2nd IA:
							0.662 (95% CI: 0.518, 0.845; *p* <0.001)	0.521 (95% CI: 0204, 1.326; *p =* 0.171)
				M16-043	Percentage of Participants with CR and Cri per Investigator assessment	*M16-043*		
				(ClinicalTrials.gov ID: NCT03069352, Phase 3)		211 (143 [including 18 Japanese] in the venetoclax + LDC, vs. 68 [including 9 Japanese] in placebo + LDC)	Percentage of Participants with CR and Cri:	Percentage of Participants with CR and Cri:
					*M16-043*		(95% CI: 57.0, 73.0; *p*< 0.001)	(95% CI: 44.7, 84.4; *p =*0.001)
					OS			
							*M16-043*	*M16-043*
							OS in the PA:	OS in the PA:
							0.749 (95% CI: 0.524, 1.071; *p* = 0.114)	**1.169** (95% CI: 0.468, 2.923; *p* = 0.735)
							***Not statistically significant***	
							OS in additional analysis 6 months after the PA:	OS in additional analysis 6 months after the PA:
Vidaza/Azacitidine	Partial change (new indication)						0.704 (95% CI: 0.503, 0.985; *p* = 0.040)	0.928 (95% CI: 0.399, 2.156; p = 0.864)
	*Expedited review*							
Keytruda/Pembrolizumab (Genetical Recombination)	Partial change (new indication, new dosage)	Aug-21	Inoperable or recurrent PD-L1 positive, hormone receptor-negative, HER2-negative breast cancer	KEYNOTE-355	PFS per BICR assessment in the following population:	847 in Part 2 (566 [including 61 Japanese] in the pembrolizumab + IC, vs. 281 [including 26 Japanese] in the placebo + IC)	PFS in PD-L1 CPS ≥ 10:	PFS in PD-L1 CPS ≥ 10:
				(ClinicalTrials.gov ID: NCT02819518, Phase 3)	1. ITT		0.65 (95% CI: 0.49, 0.86; *p* = 0.0012)	0.52 (95% CI: 0.20, 1.34; *p* = 0.0794)
					2. PD-L1 CPS ≥ 1		PFS in PD-L1 CPS ≥ 1:	PFS in PD-L1 CPS ≥ 1:
					3. PD-L1 CPS ≥ 10		0.74 (95% CI: 0.61, 0.90; *p* = 0.0014)	0.62 (95% CI: 0.35, 1.09; *p* = 0.0480)
							***Not statistically significant***	
					OS in the following population:			
					1. ITT		PFS in ITT:	PFS in ITT:
					2. PD-L1 CPS ≥ 1		0.82 (95% CI: 0.69, 0.97; *p* = 0.0112, No formal statistical test was performed)	0.64 (95% CI: 0.38, 1.07; p = 0.0416)
					3. PD-L1 CPS ≥ 10		OS in PD-L1 CPS ≥ 10 in the 2nd IA:	OS in PD-L1 CPS ≥ 10 in the 2nd IA:
							0.69 (95% CI: 0.51, 0.93; p = 0.0066)	0.47 (95% CI: 0.16, 1.36; *p* = 0.0769)
							***Not statistically significant***	
							*** OS in PD-L1 CPS ≥ 1 in the 2nd IA:***	OS in PD-L1 CPS ≥ 1 in the 2nd IA:
							0.82 (95% CI: 0.68, 1.00; p = 0.0263)	0.51 (95% CI: 0.28, 0.95; *p* = 0.0161)
							***Not statistically significant***	
							OS in ITT in the 2nd IA:	OS in ITT in the 2nd IA:
							*** 0.87 (95% CI: 0.73, 1.03; p = 0.0579, No formal statistical test was performed)***	0.47 (95% CI: 0.27, 0.82; p = 0.0031)
-/Carboplatin	Partial change (new dosage)		Breast cancer					
Abraxane/Paclitaxel	Partial change (new dosage)							
Keytruda/Pembrolizumab (Genetical Recombination)	Partial change (new indication)	Aug-21	Unresectable, advanced or recurrent MSI-High Colorectal Carcinoma	KEYNOTE-177	PFS per BICR assessment	307 (153 [including 12 Japanese] in the pembrolizumab, vs. 154 [including 10 Japanese] in the IC)	PFS in the final analysis:	PFS in the final analysis:
				(ClinicalTrials.gov ID: NCT02563002, Phase 3)	OS		0.60 (95% CI: 0.45, 0.80; *p* = 0.0002)	0.46 (95% CI: 0.14, 1.55; *p* = 0.0972)
							OS in the 2nd IA:	OS in the 2nd IA:
							0.77 (95% CI: 0.54, 1.09; *p* =0.0694)	0.53 (95% CI: 0.13, 2.23; *p* = 0.1902)
							***Not statistically significant***	
Opdivo/Nivolumab (Genetical Recombination)	Partial change (new dosage)	Aug-21	Unresectable or metastatic renal cell carcinoma	ONO-4538-81/CA2099ER	PFS per BICR assessment	651 (323 [including 22 Japanese] in the nivolumab + cabozantinib, vs. 328 [including 24 Japanese] in the sunitinib)	PFS:	PFS:
				(ClinicalTrials.gov ID: NCT03141177, Phase 3)			0.51 (95% CI: 0.41, 0.64; *p* <0.0001)	**1.68** (95% CI: 0.54, 5.21)
Cabometyx/Cabozantinib Malate	Partial change (new dosage)							
Opdivo/Nivolumab (Genetical Recombination)	Partial change (new indication, new dosage)	Nov-21	Unresectable, advanced or recurrent gastric cancer	ATTRACTION-4(ClinicalTrials.gov ID: NCT02746796, Phase 2/3)	PFS per BICR assessment	724 in Part 2 (362 [including 198 Japanese] in the nivolumab + SOC or CAPOX, vs. 362 [including 197 Japanese] in SOC or CAPOX)	PFS:	PFS:
							0.68 (95% CI: 0.54, 0.85; *p* = 0.0007)	0.83 (95% CI: 0.62, 1.13)
					OS		OS in the final analysis:	OS in the final analysis:
							0.90 (95% CI: 0.75, 1.08; p = 0.257)	**1.04**(95% CI: 0.81,1.32)
							***Not statistically significant***	1.04 (95% CI: 0.81, 1.32)
				CheckMate649 (ClinicalTrials.gov ID: NCT02872116, Phase 3)	PFS per BICR assessment in PD-L1 CPS ≥ 5 population	1,581 (789 [including 57 Japanese] in the nivolumab + ICC, vs. 792 [including 52 Japanese] in ICC)	PFS in PD-L1 CPS ≥ 5:	PFS in PD-L1 CPS ≥ 5:
							0.68 (95% CI: 0.58, 0.79; *p* < 0.0001)	**1.13** (95% CI: 0.54–2.37)
					OS in PD-L1 CPS ≥ 5 population			
							OS in PD-L1 CPS ≥ 5 in the IA:	OS in PD-L1 CPS ≥ 5 in the IA:
							0.71 (95% CI: 0.61, 0.83; *p* < 0.0001)	**1.08** (95% CI: 0.52, 2.24)
Opdivo/Nivolumab (Genetical Recombination)	Partial change (new indication, new dosage)	Nov-21	Adjuvant therapy of esophageal cancer	ONO-4538-43/CA209-577 (ClinicalTrials.gov ID: NCT02743494, Phase 3)	DFS per investigator assessment	794 (532 [including 50 Japanese] in the nivolumab, vs. 262 [including 13 Japanese] in the placebo)	DFS:	DFS:
							0.69 (95% CI: 0.56, 0.85; *p* = 0.0003)	**1.36** (95% CI: 0.56, 3.32)
Verzenio/Abemaciclib	Partial change (new indication, new dosage)	Dec-21	Adjuvant therapy for hormone receptor-positive, HER2-negative breast cancer at high risk of recurrence	monarchE (ClinicalTrials.gov ID: NCT03155997, Phase 3)	IDFS per investigator assessment	5,637 (2,808 [including 181 Japanese] in the abemaciclib + endocrine, vs. 2,829 [including 196 Japanese] in the endocrine)	IDFS in the 2nd IA:	IDFS in the 2nd IA:
							0.747 (95% CI: 0.598, 0.932; *p* = 0.00957)	**1.282** (95% CI: 0.568, 2.892)
Keytruda/Pembrolizumab (Genetical Recombination)	Partial change (new indication, new dosage)	Dec-21	Unresectable advanced or recurrent endometrial cancer that has progressed following prior chemotherapy	KEYNOTE-775/E7080-309	PFS per BICR assessment	827 (411 [including 52 Japanese] in the Lenvatinib + pembrolizumab, vs. 416 [including 52 Japanese] in the IC)	PFS in Non-MSI-high:	PFS in Non-MSI-high:
	*Orphan drug*			(ClinicalTrials.gov ID: NCT03517449, Phase 3)	OS		0.60 (95% CI: 0.50, 0.72; *p* < 0.0001)	**1.04** (95% CI: 0.63, 1.73; *p* = 0.5646)
							PFS in ITT:	PFS in ITT:
							0.56 (95% CI: 0.47, 0.66; *p* < 0.0001)	0.81 (95% CI: 0.50, 1.31; *p* = 0.1961)
							OS in Non-MSI-high in the 1st IA:	OS in Non-MSI-high in the 1st IA:
							0.68 (95% CI: 0.56, 0.84; *p* = 0.0001)	0.74 (95% CI: 0.41, 1.34; *p* = 0.1610)
							OS in ITT in the 1st IA:	OS in ITT in the 1st IA:
Lenvima/Lenvatinib Mesilate	Partial change (new indication, new dosage)						0.62 (95% CI: 0.51, 0.75; *p* < 0.0001)	0.59 (95% CI: 0.33, 1.01; *p* = 0.0314)
	*Orphan drug*							
Opdivo/Nivolumab (Genetical Recombination)	Partial change (new indication, new dosage)	Mar-22	Adjuvant treatment for urothelial carcinoma	CA209-274	DFS per investigator assessment in the following population:	709 (353 [including 27 Japanese] in the nivolumab, vs. 356 [including 22 Japanese] in the placebo)	DFS in ITT:	DFS in ITT:
				(ClinicalTrials.gov ID: NCT02632409, Phase 3)	1. ITT		0.70 (95% CI: 0.57, 0.86; *p* = 0.0008)	0.77 (95% CI: 0.35, 1.69)
							DFS in PD-L1 ≥ 1%:	DFS in PD-L1 ≥ 1%:
					2. PD-L1 ≥ 1%		0.55 (95% CI: 0.39, 0.77; *p* = 0.0005)	**1.10** (95% CI: 0.31, 3.92)
Imjudo/Tremelimumab (Genetical Recombination)	Initial	Dec-22	Unresectable advanced or recurrent non-small cell lung cancer	POSEIDON	PFS per BICR assessment	1,013 (338 [including 21 Japanese] in the tremelimumab + durvalumab + chemotherapy (T/D/C), vs. 337 [including 21 Japanese] in the durvalumab + chemotherapy (D/C), vs. 337 [including 28 Japanese] in the chemotherapy)	PFS in the final analysis:	PFS in the final analysis:
				(ClinicalTrials.gov ID: NCT03164616, Phase 3)	OS		T/D/C:	T/D/C:
							0.72 (95% CI: 0.600, 0.860; *p* = 0.00031)	0.52 (95% CI: 0.243, 1.081)
							D/C:	D/C:
							0.74 (95% CI: 0.620, 0.885; *p* = 0.00093)	NR
							OS in the final analysis:	OS in the final analysis:
							T/D/C:	T/D/C:
							0.77 (95% CI: 0.650, 0.916; *p* = 0.00304)	0.86 (95% CI: 0.431, 1.670)
							D/C:	D/C:
							0.86 (95% CI: 0.724, 1.016; *p* = 0.07581)	NR
Imfinzi/Durvalumab (Genetical Recombination)	Partial change (new indication, new dosage)						***Not statistically significant***	
Keytruda/Pembrolizumab (Genetical Recombination)	Partial change (new indication, new dosage)	May-24	Curatively unresectable biliary tract cancer	KEYNOTE-966	OS	1,069 (533 [including 58 Japanese] in the pembrolizumab, vs. 536 [including 44 Japanese] in the placebo)	OS in the final analysis:	OS in the final analysis:
				(ClinicalTrials.gov ID: NCT04003636, Phase 3)			0.83 (95% CI: 0.72, 0.95; *p* = 0.0034)	**1.08** (95% CI: 0.70, 1.68)
Datroway/Datopotamab Deruxtecan (Genetical Recombination)	Initial	Dec-24	Unresectable or recurrent hormone receptor-positive, HER2-negative breast cancer who have received prior chemotherapy	TROPION-Breast01 (ClinicalTrials.gov ID: NCT05104866, Phase 3)	PFS per BICR assessment	732 (365 [including 32 Japanese] in the datopotamab deruxtecan, vs. 367 [including 38 Japanese] in the ICC)	PFS:	PFS:
					OS		0.63 (95% CI: 0.52, 0.76; *p* < 0.0001)	0.79 (95% CI: 0.45, 1.37)
							OS in the 1st IA:	OS in the 1st IA:
							0.84 (95% CI: 0.62, 1.14; *p* = 0.2615)	**1.49** (95% CI: 0.53, 4.51)
							***Not statistically significant***	
							OS in the 2nd IA:	OS in the 2nd IA:
							0.93 (95% CI, 0.76, 1.13; *p* = 0.4712)	NR
							***Not statistically significant***	
Imfinzi/Durvalumab (Genetical Recombination)	Partial change (new indication, new dosage)	Mar-25	Maintenance therapy following definitive chemoradiotherapy for limited-stage small cell lung cancer	ADRIATIC	PFS per BICR assessment	530 (264 [including 19 Japanese] in the durvalumab, vs. 266 [including 31 Japanese] in the placebo)	PFS:	PFS:
	*Orphan drug*			(ClinicalTrials.gov ID: NCT03703297, Phase 3)			0.76 (95% CI: 0.606, 0.950; *p* = 0.01608)	**1.05** (95% CI: 0.440, 2.362)
					OS		OS in the 1st IA:	OS in the 1st IA:
							0.73 (95% CI: 0.569, 0.928; *p* = 0.01042)	0.67 (95% CI: 0.239,1.624)

BICR: blinded independent central review; CAPOX: capecitabine + oxaliplatin; CI: Confidence Interval; CPS: combined positive score; CR: Complete Remission; Cri: Complete Remission With Incomplete Marrow Recovery; DFS: disease-free survival; HR: hazard ratio; IA: interim analysis; IC: investigator’s choice; ICC: investigator’s choice chemotherapy; ID: identifier; IDFS: invasive disease-free survival; IRC: independent response committee; IRF: independent review facility; ITT: Intent-to-Treat; LDC: Low-dose cytarabine; MSI-high: microsatellite instability-high; NCT: National Clinical Trials; ND: not disclosed; NR: not reported; OS: overall survival; PA: primary analysis; Pd: combination of pomalidomide and dexamethasone; PD-L1: programmed cell death 1; PFS: progression-free survival; PMDA: Pharmaceuticals Medical Devices Agency; SOC: standard of care; vs.: versus; %: percentage

Bold font in the table indicates results that are not statistically significant.

**Fig. 2:**
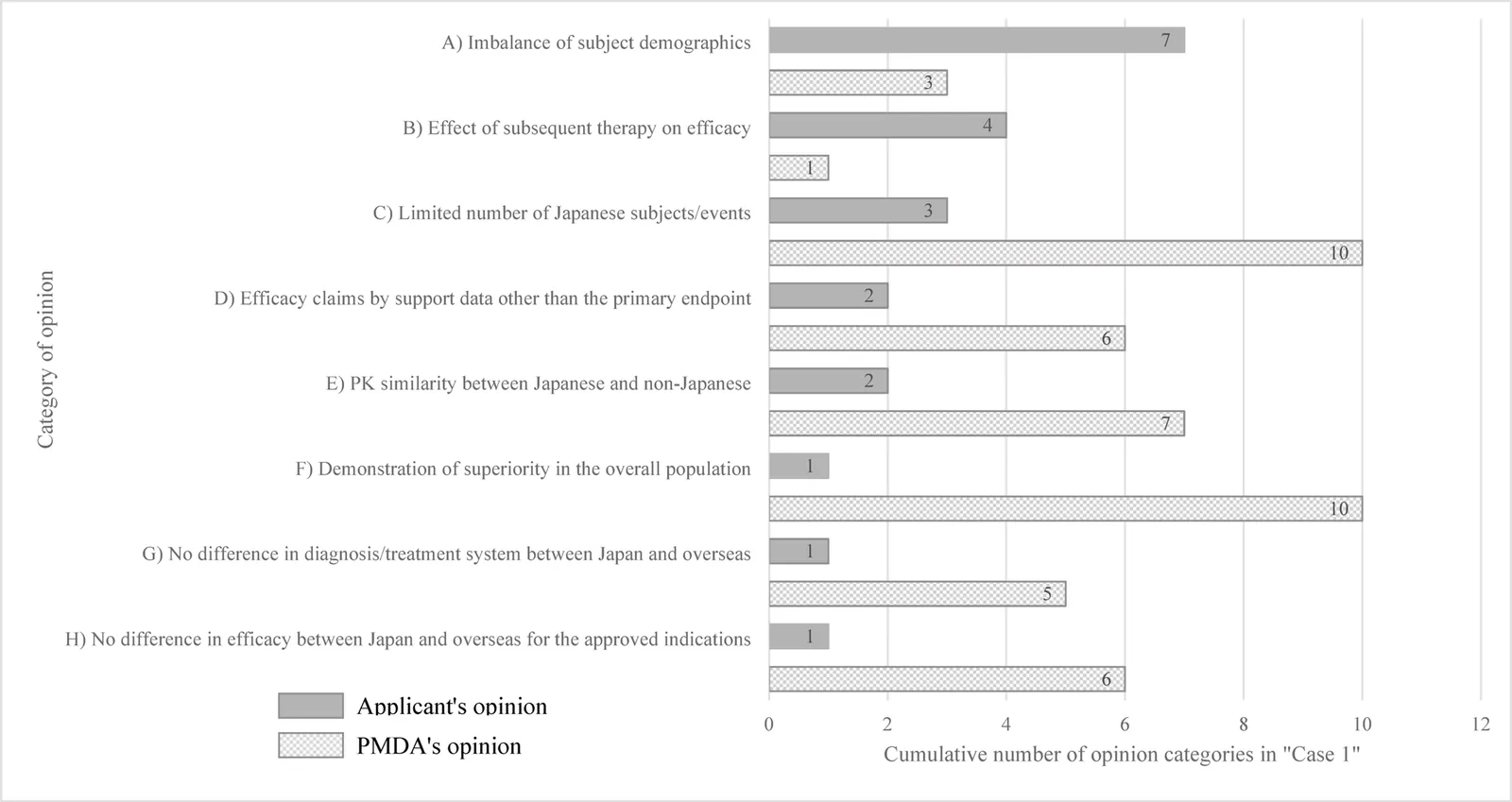
Trend of the Applicant’s and PMDA’s opinion on the primary results in Japanese subpopulation discussed in the PMDA assessment report. *Note*: If multiple reasons are described in the PMDA review report, multiple opinion categories (A-H) may apply to a single drug

Japanese subgroup analyses were a central feature of the PMDA assessments. Notably, in 7.9% (13/165) of the approvals, efficacy results for the primary endpoints such as OS, PFS, invasive disease-free survival (IDFS), or disease-free survival (DFS) in Japanese population were not consistent with HR > 1.0 compared to the overall population, which is classified as Case 1 by the research definition. It was evident from the PMDA assessment reports that the reasons for the differences in the results between the Japanese subgroup and the overall population were discussed in detail between the applicant and the PMDA through information requests during the review process. In many cases, the applicant explained the reason for the difference in the results between the Japanese subgroup and the overall population using the results of additional sub-analyses. The applicant’s opinion on the reason for the results specific to the Japanese population and the points of discussion and interpretation by the PMDA described in the PMDA assessment report were tabulated by each approval case (Table 3). Based on the information in (Table 3), we examined whether there were differences between the applicant’s and PMDA’s perspectives and the rationale for efficacy in Japanese patients when the primary efficacy endpoints showed different results between the Japanese subpopulation and the overall population (Fig. 2). From the 13 approvals in Case 1, the applicants tended to explain the efficacy in Japanese population mainly based on the data including the subgroup analysis of the pivotal trial. On the other hand, it was noteworthy that the PMDA highlighted limitations due to the small number of Japanese participants and events, and they tended to refer to the efficacy in the Japanese population by making maximum use of existing data outside the pivotal trial in addition to the overall pivotal trial results.

**Table 3:** Applicant/PMDA’s discussion and interpretation for drugs with discrepancy in Japanese subpopulation (Case 1)

Brand name/ Generic name	Type of cancer	Percentage of Japanese participants in the trial	Efficacy endpoint in Japanese population [HR]	Opinion giver	Classification of reasons for claims of efficacy in Japanese							
					A	B	C	D	E	F	G	H
Sarclisa/Isatuximab	Multiple myeloma	EFC14335 4.2%	PFS 1.2	Applicant			X					
				PMDA					X	X	X	
Tesentriq/Atezolizumab Avastin/Bevacizumab	Hepatocellular carcinoma	IMbrave150 12.3%	OS 1.71	Applicant	X				X		X	
				PMDA			X	X		X		X
Braftovi/Encorafenib Mektovi/Binimetinib	Colorectal cancer	BEACON CRC 3.0% in Ph3 part	OS 1.58	Applicant								
				PMDA			X	X	X	X	X	
Venclexta/Venetoclax Vidaza/Azacitidine	Acute myeloid leukemia	M16-043 12.8%	OS 1.169	Applicant	X	X	X	X				
				PMDA			X	X	X	X		
Opdivo/Nivolumab Cabometyx/Cabozantinib Malate	Renal cell carcinoma	ONO-4538-81 7.1%	PFS 1.68	Applicant	X	X	X					
				PMDA			X		X	X	X	X
Opdivo/Nivolumab	Gastric cancer	ATTRACTION-4 54.6% CheckMate649 6.9%	ATTRACTION-4 OS 1.04 CheckMate649 PFS 1.13, OS 1.08	Applicant		X			X	X		X
				PMDA		X			X	X		X
Opdivo/Nivolumab	Esophageal cancer	ONO-4538-43 7.9%	DFS 1.36	Applicant	X							
				PMDA	X			X	X	X	X	X
Verzenio/Abemaciclib	Breast cancer	monarchE 6.7%	IDFS 1.282	Applicant				X				
				PMDA			X	X				
Keytruda/Pembrolizumab Lenvima/Lenvatinib	Endometrial cancer	KEYNOTE-775 12.6%	PFS 1.04	Applicant	X							
				PMDA	X		X					
Opdivo/Nivolumab	Urothelial carcinoma	CA209-274 6.9%	DFS 1.10	Applicant	X							
				PMDA	X		X	X		X		X
Keytruda/Pembrolizumab	Biliary Tract Cancer	KEYNOTE-966 9.5%	OS 1.08	Applicant	X	X		X				
				PMDA			X		X		X	X
Datroway/Datopotamab Deruxtecan	Breast cancer	TROPION-Breast01 9.6%	OS 1.49	Applicant		X						
				PMDA			X			X		
Imfinzi/Durvalumab	Small Cell Lung Cancer	ADRIATIC 9.4%	PFS 1.05	Applicant								
				PMDA			X			X		

DFS: disease-free survival; HR: hazard ratio; IDFS: invasive disease-free survival; OS: overall survival; PFS: progression-free survival; PMDA: Pharmaceuticals Medical Devices Agency

Classification of reasons for claims of efficacy in Japanese:

(A) Imbalance of subject demographics, (B) Effect of subsequent therapy on efficacy, (C) Limited number of Japanese participants/events, (D) Efficacy claims by support data other than the primary endpoint [e.g., co-primary or secondary], (E) PK similarity between Japanese and non-Japanese, (F) Demonstration of superiority in the overall population, (G) No difference in diagnosis/treatment system between Japan and overseas, (H) No difference in efficacy between Japan and overseas for the approved indications

Of the 13 approvals of Case 1 analyzed in (Table 3), the efficacy results of the Japanese subgroup analysis in the Japanese package insert were presented for only 1 approval shown below.

#### Atezolizumab plus Bevacizumab for Hepatocellular Carcinoma (Approved in September 2020)

In the IMBrave150 study, there was inconsistency in the data between the overall population and the Japanese subpopulation. Efficacy results in Japanese subgroup data were also provided in the section of CLINICAL TRIALS of Japan package insert along with that of the overall population. The reason why the results of subgroup analysis of Japanese subpopulation were added in this case could not be confirmed from the PMDA assessment report.

Of the 13 approvals for Case 1, 11 were considered tolerable in the PMDA assessment reports because there was no difference in safety between Japan and overseas and the safety profile was similar to the previously approved indications. On the other hand, all-case post-marketing surveillance were required for the 2 approvals for which differences in safety were observed between Japan and overseas (Isatuximab for multiple myeloma, Venetoclax and Azacidine for acute myeloid leukemia). The research focused on the impact of efficacy results on the approval in Japan and the impact of safety profile was outside the scope of this research. However, these findings also make it evident that regulatory discussion and approval decisions are influenced by multifactorial and complex evaluation.

### Endpoints in Pivotal Trials: OS, PFS, and Alternative Measures

OS was designed as the primary efficacy endpoint as a sole primary endpoint in 9.1% (15/165) and as co-primary or dual primary endpoints in 10.3% (17/165) of all approvals. In scenarios where OS was impractical such as indolent malignancies, high crossover rates, or rapidly evolving standards of care alternative endpoints such as PFS, IDFS or DFS were accepted.

In 4.2% (7/165) of the approvals, efficacy results for the primary endpoint of OS in the overall population did not demonstrate statistical significance, which is classified as Case 2 by the research definition. The list of trials that the primary endpoint of OS did not achieve statistical significance were tabulated (Table 4). For these 7 approvals, the PMDA emphasized the totality of evidence, including efficacy outcome in other endpoints such as PFS, and the lack of a clear trend towards shorter OS compared to the control, when rendering approval decisions. Of the 7 approvals which did not achieve the primary endpoint of OS in (Table 4), 4 approvals were identified to have restrictions in the Japan package insert, such as approval only for a part of the target population expected to have clinical benefit. The corresponding actions implemented in the Japan package insert for these 4 cases are summarized below.

**Table 4:** List of trials with OS as primary endpoint not achieving statistical significance (Case 2)

Brand name/ Generic name	Type of cancer	Percentage of Japanese participants in the trial	Study results with Not statistically significant in primary endpoints [HR (95% CI; *p* value)]	Primary rationale for PMDA determination of efficacy	Impact on Japan package insert
Keytruda/Pembrolizumab	Esophageal squamous cell carcinoma	KEYNOTE-181 24.2%	*Dual Primary Endpoints* OS by analysis population PD-L1 CPS ≥ 10: 0.70 (95% CI: 0.52,0.94; *p* = 0.00855) Squamous cell carcinoma: 0.77 (95% CI: 0.63, 0.96; *p* = 0.00894) ITT: 0.89 (95% CI: 0.75, 1.05; *p* = 0.08431)	Results of sensitivity analyses in the subgroups (PD-L1 expression, histological type) stratified by race, ECOG PS, and prior treatment with a taxane were consistent with the pre-specified analysis results. Efficacy results in the Japanese subgroups with CPS ≥ 10 and squamous cell carcinoma showed similar trends to those in the overall population.	Approved for use in narrower patient segment than those in KEYNOTE-181 Patient with PD-L1 CPS ≥ 10 and squamous cell carcinoma of the esophagus
Venclexta/Venetoclax Vidaza/Azacitidine	Acute myeloid leukemia	M16-043 12.8%	*A Sole Primary Endpoint* OS 0.749 (95% CI: 0.524, 1.071; *p* = 0.114)	OS (dual primary endpoint) in another pivotal study (M15-656) demonstrated superiority in venetoclax plus azacitidine over placebo plus azacitidine. CR + CRi rate in venetoxlax plus LDAC tended to be higher compared to placebo plus LDAC in M16-043 study. No clear difference was observed in the PK, efficacy between Japanese and non-Japanese.	No impact observed
Keytruda/Pembrolizumab	MSI-High Colorectal Carcinoma	KEYNOTE-177 7.2%	*Dual Primary Endpoints* OS 0.77 (95% CI: 0.54, 1.09; *p* =0.0694)	PFS (dual primary endpoint) in pembrolizumab demonstrated superiority over IC and showed a clinically significant effect. OS (dual primary endpoint in the study) did not tend to be shorter in pembrolizumab than in IC. No clear difference was observed in the efficacy between Japanese and overall population.	No impact observed
Keytruda/Pembrolizumab -/Carboplatin Abraxane/Paclitaxel	Breast cancer	KEYNOTE-355 10.3%	*Dual Primary Endpoints* PFS in PD-L1 CPS ≥ 1 0.74 (95% CI: 0.61, 0.90; *p* = 0.0014) OS by PD-L1 CPS CPS ≥ 10: 0.69 (95% CI: 0.51, 0.93; *p* = 0.0066) CPS ≥ 1: 0.82 (95% CI: 0.68, 1.00; *p* = 0.0263)	PFS (dual primary endpoint) in the PD-L1-positive (CPS ≥ 10) population in pembrolizumab plus IC demonstrated superiority over placebo plus IC, and a clinically significant effect was observed. OS (dual primary endpoint in the study) in the PD-L1-positive (CPS ≥ 10) population tended to be longer in pembrolizumab plus IC than in the placebo plus IC. Results in the Japanese population did not tend to be clearly different from those in the overall population.	Only approved for use in patients with PD-L1 CPS ≥ 10
Opdivo/Nivolumab	Gastric cancer	ATTRACTION-4 54.6%	*Dual Primary Endpoints* OS 0.90 (95% CI: 0.75, 1.08; *p* = 0.257)	Another pivotal study (CheckMate649) demonstrated the efficacy of nivolumab plus IC in prolonging OS (co-primary endpoint). ATTRACTION-4 and CheckMate649 demonstrated the superiority of nivolumab plus IC to control (placebo plus IC or IC) in PFS and a certain degree of clinically significant effect. Nivolumab plus IC was not clearly inferior to the control (placebo plus IC or IC) in OS in the overall population of ATTRACTION-4 or the Japanese population of CheckMate649.	Failure to achieve OS in ATTRACTION-4 had no impact on the package insert. Actions to be taken based on the efficacy results of another pivotal study (CheckMate649) Provided for efficacy results by PD-L1 CPS category alongside primary results, and added precautionary statement about it
Imjudo/Tremelimumab Imfinzi/Durvalumab	Non-small cell lung cancer	POSEIDON 6.9%	*Dual Primary Endpoints* OS in the durvalumab + chemotherapy 0.86 (95% CI: 0.724, 1.016; *p* = 0.07581)	POSEIDON demonstrated a statistically significant prolongation of OS in the tremelimumab/durvalumab/chemotherapy compared to chemotherapy (secondary objective). No clear differences between Japanese and non-Japanese in the PK of durvalumab and tremelimumab or in the diagnostic and therapeutic systems for patients with NSCLC in Japan and overseas. No clear differences between Japanese and non-Japanese in the efficacy of durvalumab for the approved indications.	Only approved for the regimen evaluated as secondary endpoint in the clinical trial
Datroway/Datopotamab Deruxtecan	Breast cancer	TROPION-Breast01 9.6%	*Dual Primary Endpoints* OS 1st IA: 0.84 (95% CI: 0.62, 1.14; *p* = 0.2615) 2nd IA: 0.93 (95% CI, 0.76, 1.13; *p* = 0.4712)	PFS (dual primary endpoint) demonstrated superiority of datopotamab deruxtecan over the ICC and showed a clinically significant effect. OS (dual primary endpoint) showed no trend towards shorter in datopotamab deruxtecan compared with the ICC. PFS in Japanese population did not tend to differ from the results from the overall population.	No impact observed

CI: Confidence Interval; CPS: combined positive score; CR: complete remission; Cri: complete remission with incomplete blood count recovery; ECOG PS: Eastern Cooperative Oncology Group performance status; HR: hazard ratio; IA: interim analysis; IC: Investigator’s choice of standard of care; ICC: Investigator’s choice of Chemotherapy; ITT: Intent-to-Treat; LDAC: low dose cytarabine; MSI-high: microsatellite instability-high; NSCLC: Non-Small Cell Lung Cancer; OS: overall survival; PD-L1: programmed cell death 1; PFS: progression-free survival; %: percentage

#### Pembrolizumab for Esophageal Squamous Cell Carcinoma (Approved in August 2020)

In the KEYNOTE-181 study, the results for OS in the population (a: patients with programmed cell death 1 [PD-L1] combined positive score [CPS] ≥ 10, b: patients with squamous cell carcinoma, c: Intent-to-Treat [ITT]), which were the dual primary endpoints, failed to meet the prespecified criteria. However, the OS in the pembrolizumab tended to be longer than that in the Investigator’s choice (IC) in 2 primary analysis sets: (a) patients with PD-L1 CPS ≥ 10 and (b) patients with squamous cell carcinoma, and the result for OS in these analysis sets appeared to differ from the result in the ITT population. PMDA further reviewed the efficacy of pembrolizumab by PD-L1 expression status and by histology in subsections (a) and (b), and they concluded that the results of KEYNOTE-181 study demonstrated a certain level of efficacy of pembrolizumab in the treatment of chemotherapy-treated patients with radically unresectable, advanced or recurrent PD-L1 CPS ≥ 10 esophageal squamous cell carcinoma. PMDA judged that the percentage of PD-L1 CPS in patients who responded to pembrolizumab in KEYNOTE-181 study should be included in the section of CLINICAL TRIALS of Japan package insert, and the following precautionary statement should be included in the “Precautions Concerning Indication” section. PMDA thus approved pembrolizumab for the indication of a more limited patient population than that in KEYNOTE-181 study.

- Pembrolizumab should be administered to patients who have been demonstrated to have a tumor with PD-L1 expression by a highly experienced pathologist or at a laboratory facility using the approved in vitro diagnostic or medical device, after carefully reading the “CLINICAL TRIALS” section to understand the percentage of PD-L1 CPS.

#### Pembrolizumab Plus Carboplatin and Paclitaxel for Breast Cancer (Approved in August 2021)

At the start of the KEYNOTE-355 study, PFS assessed by the blinded independent central review (BICR) and OS in ITT population and in PD-L1 CPS ≥ 1 population were set as the dual primary endpoint in part 2 of the trial. PD-L1 CPS ≥ 10 population was added to the primary analysis set according to the protocol amendment after the first interim analysis of OS, a total of 4 analyses were planned (the first analysis was the interim analysis of both OS and PFS, the second analysis was the interim analysis of OS and the final analysis of PFS, the third analysis was the interim analysis of OS, and the final analysis of OS). The superiority of pembrolizumab in combination with IC (pembrolizumab/IC) to the placebo plus IC (placebo/IC) was demonstrated in the PD-L1 CPS ≥ 10 population in the primary endpoint of PFS. In the PD-L1 CPS ≥ 1 population, which was tested according to a hierarchical procedure, the superiority of pembrolizumab/IC was not demonstrated for PFS. In the second interim analysis of OS, the other primary endpoint, the superiority of pembrolizumab/IC to the placebo/IC was not demonstrated in the PD-L1 CPS ≥ 10 population or in the PD-L1 CPS ≥ 1 population, which was tested according to the test procedure and alpha allocation specified in the trial. The discussion on efficacy in the PMDA assessment report focused only on the CPS ≥ 10 population. Based on the results of KEYNOTE-355 study, PMDA concluded that the efficacy of pembrolizumab was demonstrated in terms of the following points, and they approved the indication for the population only with providing trial results of the population only in the section of CLINICAL TRIALS of Japan package insert.

- The superiority of pembrolizumab/IC over placebo/IC was demonstrated in PFS in the PD-L1 CPS ≥ 10 population, one of the primary endpoints, and the magnitude of the effect was clinically significant.
- OS in the PD-L1 CPS ≥ 10 population, one of the primary endpoints, tended to be longer in pembrolizumab/IC than in the placebo/IC.

In light of the description in the PMDA assessment report, the fact that the superiority in OS was not demonstrated in the population with PD-L1 CPS ≥ 10 did not significantly affect the approval.

#### Nivolumab Plus Chemotherapy (CAPOX, SOX, or FOLFOX6) for Gastric Cancer (Approved in November 2021)

The ATTRACTION-4 study, one of the pivotal trials, for this approval demonstrated the superiority of nivolumab in combination with IC (CAPOX: capecitabine plus oxaliplatin, SOX: oxaliplatin plus S-1)over IC plus placebo in the primary endpoint of PFS, while the dual primary endpoint of OS did not achieve statistical significance. The CheckMate649 study, another pivotal trial, demonstrated the superiority of nivolumab in combination with IC (CAPOX, FOLFOX6: fluorouracil, leucovorin plus oxaliplatin) over IC in the Co-primary endpoints of PFS and OS in the CPS ≥ 5 population. Furthermore, a statistically significant improvement in OS was observed in the nivolumab/IC compared with the IC in both the CPS ≥ 1 population and the ITT population, which were key secondary endpoints. In the overall population of the ATTRACTION-4 study and the Japanese population of the CheckMate649 study, OS in the nivolumab/IC was not clearly inferior to that in the control. Therefore, it is considered that these results did not affect the approval itself. However, as the efficacy of nivolumab/IC tended to differ depending on PD-L1 expression status, PMDA requested an additional evaluation of the efficacy by PD-L1 expression status (CPS cutoff values 1 and 5). Based on the following points, PMDA instructed the applicant to include the result of exploratory analysis of nivolumab/IC in efficacy by PD-L1 expression status in CheckMate649 study in the section of CLINICAL TRIALS and to include a precautionary statement in the Japan package insert regarding the necessity for clinical judgement on concomitant use with nivolumab based on the PD-L1 expression rate.

- The PD-L1 CPS ≥ 5 population was selected as the primary analysis population in the CheckMate649 study, taking into account that the applicant suggested that the PD-L1 expression status based on CPS may be a predictor of response to nivolumab.
- The results of the CheckMate649 study showed that the add-on effect of nivolumab to chemotherapy tended to be smaller in the 1 ≤ CPS < 5 and CPS < 1 population than in the CPS ≥ 5 population.
- The incidence of adverse events tended to be higher in the nivolumab/IC than in the control in the CheckMate649 study and the ATTRACTION-4 study.

#### Tremelimumab Plus Durvalumab for Non-small Cell Lung Cancer (Approved in December 2022)

At the start of the POSEIDON study, PFS assessed by BICR was set as the primary endpoint of this trial, and the combination therapy with tremelimumab, durvalumab and chemotherapy (T/D/Chemo) and the combination therapy with durvalumab and chemotherapy (D/Chemo) were to be compared with the Chemotherapy (Chemo). However, due to the protocol amendment, only the comparison between the D/Chemo and the Chemo was finally set as the primary objective of this trial, and the primary endpoints were changed to PFS and OS (dual primary endpoints). In the primary analysis, the superiority of the D/Chemo to the Chemo was demonstrated for PFS, but the superiority of the D/Chemo to the Chemo was not demonstrated for OS. On the other hand, the comparison between the T/D/Chemo and Chemo, the secondary objectives, showed statistically significant prolongation of PFS and OS in the T/D/Chemo compared with the Chemo. Based on the results, PMDA concluded that the efficacy of T/D/Chemo was demonstrated and approved only the regimen different from the treatment group specified for the primary objective, and only the results of the T/D/Chemo in POSEIDON trial were presented in the section of CLINICAL TRIALS.

## Discussion

### Regulatory Context and Importance of OS

In Japan, the development and approval of anticancer drugs have historically emphasized OS as a key efficacy endpoint. This principle is clearly articulated in the revised guideline of anticancer drug [8]. The guideline underscores OS as the most clinically meaningful measure of benefit, reflecting its direct relevance to patient outcomes. However, the evolving landscape of oncology drug development, characterized by innovative trial designs and targeted therapies, has necessitated a more nuanced approach to endpoint selection.

While OS remains a cornerstone of regulatory evaluation, practical considerations such as disease characteristics, treatment settings, and feasibility constraints often preclude its use as the sole primary endpoint. Even in such cases, PMDA proposes that OS should be evaluated rigorously to ensure that survival benefit is not overlooked, even if OS is not the primary determinant of approval. However, if a trial incorporates primary endpoints such as PFS alongside OS and demonstrates superiority for the non-OS endpoint, approval in Japan has been granted even in scenarios where OS was not achieved. This approach reflects a pragmatic balance between scientific rigor and the realities of oncology drug development, particularly in global trials where endpoint strategies must accommodate diverse regulatory expectations.

### Observed Trends in Approval Decisions

Analysis of trends in approvals across indications showed no clear pattern of correlation between the magnitude of HR (HR close to 1.0 or not [e.g., HR cutoff < 1.1 or ≥ 1.1]) and regulatory outcomes (e.g., approval decision, impact on Japan package insert). No cancer-type-specific tendencies were observed, and the stance of regulatory discussions remained consistent across tumor types. Furthermore, orphan drug designation did not exert a discernible influence on the depth or focus of regulatory deliberations. These findings suggest that Japanese regulatory decisions are not driven by rigid numerical thresholds or categorical distinctions but rather by a holistic evaluation of clinical benefit and supporting evidence.

Interestingly, approvals were observed not only for line extensions with well-established drug profiles but also for new molecular entities, even in cases where OS was not met or HR in the Japanese subgroup tended to be different from that in the overall population. This underscores that the size of the HR alone does not constitute a critical determinant in regulatory considerations. Instead, factors such as trial design integrity, robustness of secondary endpoints, and consistency of benefit across subgroups appear to carry greater weight in decision-making.

### Role of Japanese Subgroup Data

In the PMDA reviews, efficacy and safety in the Japanese population are always discussed as part of the approval process. However, greater emphasis is placed on results from the overall global population, reflecting the integrated nature of contemporary oncology development programs. Importantly, the focus lies on whether the observed trends in Japanese patients align with those in the global population.

The evaluation of Japanese subgroup data typically centers on qualitative consistency rather than quantitative thresholds. Regulators assess whether efficacy and safety profiles in Japanese patients mirror those observed globally, considering factors such as pharmacokinetics, tolerability, and exposure-response relationships. This perspective aligns with the broader regulatory principle of extrapolation, which seeks to leverage global evidence while addressing local scientific and clinical considerations. Even if different trend in efficacy is seen between the overall population and Japanese subpopulation, PMDA generally makes a decision on approval based on the totality of evidence fully utilizing the existing data. It was confirmed from Table 3 that the evaluation by PMDA has been made in consideration of not only the data within the trials conducted but also the knowledge obtained in the approved indications. This analysis also revealed that labeling is rarely impacted in Japan even if differences in efficacy trends are observed between the Japanese population and the overall population.

## Conclusions

The findings from the analysis based on approved products provides important insights into the regulatory considerations for oncology drug approvals in Japan, particularly regarding the role of Japanese subpopulation data and OS in pivotal trials. While OS remains the most clinically meaningful endpoint, practical limitations in certain indications have led the PMDA to accept scientifically justified alternatives such as PFS. Approval decisions are not driven by rigid HR thresholds or categorical distinctions; rather, they reflect a holistic evaluation of trial design integrity, robustness of secondary endpoints, and consistency of benefit across subgroups.

Japanese subgroup data are consistently reviewed during the approval process; however, greater emphasis is placed on results from the overall global population. Differences in efficacy trends between Japanese and overall populations rarely influence approval status or labeling language, provided that qualitative consistency is maintained. This approach underscores the principle of extrapolation and the importance of leveraging global evidence while addressing local scientific considerations.

Although this study, based on publicly available PMDA documents, may not fully capture PMDA’s regulatory flexibility and requires caution in generalization of their regulatory stance, the findings from the analysis emphasized PMDA’s pragmatic approach to flexible evaluation endpoints and extrapolation. As MRCTs continue to evolve, early engagement with the PMDA and alignment on endpoint strategy will remain critical for successful regulatory submissions in Japan.

### Limitations

In this research, only approved new drugs were captured. In other words, it should be noted that products for which approval was denied, applications for which approvals were withdrawn, and applications for which approvals were abandoned in Japan were not captured. It should be considered the possibility that sponsors submit only products with high likelihood of approval in Japan. In addition, the impact of the difference in safety profiles between Japan and overseas on the approval in Japan was outside the scope of this research.

## Data Availability

No datasets were generated or analysed during the current study.
